# Effect of the Inclusion of Different Levels of Dietary Cactus (*Opuntia ficus-indica*) on Gilts’ Biochemical Parameters and Feed Intake during Lactation

**DOI:** 10.3390/ani10101881

**Published:** 2020-10-15

**Authors:** Ruy Ortiz, Manuel López, Rosa E. Pérez, Paola de la Paz Ramírez, Gerardo Ordaz

**Affiliations:** 1 Faculty of Veterinary Medicine and Zootechnics, Universidad Michoacana de San Nicolás de Hidalgo, 58130 Michoacan, Mexico; ortizprofruy@gmail.com (R.O.); mvzmanuellopez@hotmail.com (M.L.); 2 Faculty of Chemical Pharmacobiology, Universidad Michoacana de San Nicolás de Hidalgo, 58130 Michoacan, Mexico; 3 Postgraduate Student of the Faculty of Veterinary Medicine and Zootechnics, Universidad Michoacana de San Nicolás de Hidalgo, 58130 Michoacan, Mexico; pao930802@gmail.com; 4 National Center of Disciplinary Research in Animal Physiology and Genetics, INIFAP, 76280 Queretaro, Mexico; ordazog@gmail.com

**Keywords:** feed intake, animal nutrition, glucose, insulin, lactation, gilts

## Abstract

**Simple Summary:**

The digestive and metabolic adaptations suffered by sows during the late gestation-lactation transition cause a decrease in the voluntary feed intake. These conditions favor a negative energy balance, forcing the body to mobilize body reserves to meet their nutritional requirements, which represents an obstacle to expressing their reproductive and productive potential. The supplementation of the sows’ diet with cactus (*Opuntia ficus-indica* L.) improves the feed intake in lactation due to the favorable modulation of biochemical indicators. However, the optimal addition of cactus to the diet of lactating sows is unknown. In the present study, the increase in the inclusion level of cactus did not linearly increase the feed intake in lactation. However, a linear increase in plasma insulin and osteocalcin concentrations were observed, which led to a decrease in the plasma glucose concentrations. We conclude that the inclusion level of cactus (fresh base) in the diet of lactating sows of 1.0% (with respect to the body weight of the sow) could improve the feed intake in lactation and reduce the loss of body weight of the sow at weaning.

**Abstract:**

The regulation of sows’ metabolic state during the gestation-lactation transition is a requirement for a higher feed intake in lactation, an important aspect in improving animal welfare in current swine production systems. The present study aimed to evaluate the effects of the inclusion of different cactus (*Opuntia ficus-indica* L.) levels in the diet of gilts during late gestation and lactation on their biochemical parameters and voluntary feed intake during lactation. From day 85 of gestation until weaning, 40 gilts were divided into four groups: GNC (group with no cactus) with a basal diet (BD) only, G1C; group with 1% inclusion of cactus plus BD, G2C; group with 1.5% inclusion of cactus plus BD, and G3C; group with 2% inclusion of cactus plus BD. The dietary cactus supplementation increased the gilts’ feed intake (by 1.04 kg/day on average) during lactation and reduced their weight loss (4.3%) at weaning. The glucose concentrations were higher (range 73.0–83.9 mg/dL) in the GNC. The GNC had the highest triglyceride and cholesterol concentrations at day 100 of gestation. G3C had the highest osteocalcin concentration at day 100 of gestation. The highest feed intake and lowest glucose concentration were achieved with a cactus consumption of 1.04% in lactating gilts.

## 1. Introduction

The successful feeding of breeding sows does not consist only of an adequate nutrient supply in each of their productive phases (gestation or lactation). During the last few decades, the term maximizing sow feed intake has been incorporated, especially during lactation [1]. If a sow’s feed intake is not optimal, her body condition and reproductive and productive indicators after weaning are affected, irrespective of whether she is receiving an adequate supply of nutrients [2]. Research has focused on searching and evaluating food strategies that help mitigate the effects of sows’ low feed intake during lactation [3,4,5].

A low feed intake during lactation has been associated with insulin resistance in sows during the last third of gestation [3], the breastfeeding effect and the prolactin–leptin–insulin relationship [6,7], the hypergonadotropic phase post-farrowing [8], and the sow’s body condition [5,9]. Nutritional strategies which focus on the input quantity and quality have been implemented in lactating sows. Among the implemented strategies are the increased energy density of the diet [10], the modification of the protein and amino acid levels [11], which has been proposed as an ideal protein profile based on the mobilization of body reserves [12], and the addition of dietary fiber to the sows’ diets [13,14]. However, the implementation of these strategies has not alleviated completely the feed intake deficit during lactation.

It has been reported [15,16] that supplementing lactating sows’ diets with cactus (*Opuntia ficus-indica* L). increases their feed intake. The effects of this supplementation include a reduction in the sows’ glycemic index. In addition, the consumption of cactus favors a lower body weight loss at weaning, a shorter weaning-estrus interval, and a larger subsequent litter size. However, the optimal supplementation level of cactus to the diet of lactating sows has not yet been established. Therefore, the goal of this study was to evaluate the effect of the inclusion of different cactus (*O. ficus-indica* L.) levels in the diets of sows during late gestation and lactation on their biochemical parameters and voluntary feed intake during lactation.

## 2. Materials and Methods

This research was carried out at Tarímbaro, Michoacan, Mexico (19°46′ N, 101°08′ W; altitude: 1855 m). The animals used in this study were bred in accordance with the regulations of the zootechnical and zoosanitary legislation of Mexico for the humanitarian care and use of animals in research, Ministry of Agriculture and Rural Development. 

All the procedures used in this study were approved by the Animal Rights and Protection Act in the state of Michoacán of Ocampo: Volume CLXIX, Num. 58, tenth section, Chapter XII, Articles 62–64 experimentation, and by the official Mexican standard OMS-062-ZOO-1999. This research did not involve the sacrifice of animals, only the monitoring of the metabolic profiles of the gilts during different reproductive phases by blood sample collection. Thus, after the experiments, the animals were incorporated into the reproductive herd of the swine production system of the Faculty of Veterinary Medicine and Zootechnics of the Universidad Michoacana de San Nicolás de Hidalgo.

### 2.1. Animal Diets and Housing 

Forty gilts of the Yorkshire × Landrace × Pietrain genotype were used in a completely randomized design. The gilts were served (129 ± 0.43 kg of live weight) by Pietrain genotype boars who mounted them naturally and were housed in groups (*n* = 8) in 16 m^2^ pens until day 84 of gestation. From day 85 of gestation, the gilts were confined in individual pens (4.0 m^2^) until they were transferred to the farrowing and lactation room (day 110 of gestation). The gilts were housed in stainless steel cages with a plastic grid floor until weaning (21 days post-farrowing). In the farrowing and lactation room, artificial light was used between 8:00 h and 15:00 h. The temperature of the farrowing room during the experiment was between 17 and 20 °C, and a heat source was provided to each litter to guarantee the thermal comfort of the piglets (temperature between 24 and 28 °C). Farrowing was not induced, and it occurred on day 115 ± 0.13 of gestation (day 0 of lactation). The gilts at farrowing had a litter size of 13.3 ± 1.6 piglets, 12.1 ± 0.9 piglets born alive, 1.1 ± 0.3 stillbirths, and 0.2 ± 0.01 mummies. The average weight (before homogenizing litters) of the piglets at birth was 1.3 ± 0.6 kg. The litters were homogenized to 10 piglets within the first 48 h post-farrowing. The choice to evaluate only 10 piglets per litter was based on the minimum live born piglets (10 piglets) obtained. To homogenize the litters, only piglets with ≥1.2 kg live weight were considered. The piglets were supplied with commercial feed^®^ (150 g on average litter^−1^·day^−1^) from day seven post-farrowing to weaning. 

From the service day until day 85 of gestation, all the gilts received the same diet for gestating gilts (Table 1), which was 2.5 kg·day^−1^ supplied in two equal portions at 8:00 h and 16:00 h. From day 85 of gestation, the gilts were fed according to the groups established in the experimental design. There were four groups (*n* = 10 gilts·group^−1^): GNC (group with no cactus) with a basal diet (BD) only inclusion, G1C; group with 1% inclusion of cactus plus BD, G2C; group with 1.5% inclusion of cactus plus BD, and G3C; group with 2% inclusion of cactus plus BD. The cactus was supplied on a fresh basis (FB) and the amount supplied was according to the gilts’ body weight on day 85 of gestation. The cactus supply during lactation was adjusted to the body weight of the gilts on day 110 of gestation. Immediately after farrowing, all the gilts were fed a conventional diet for lactation ad libitum (Table 1). The only variant in the diet was the corresponding addition (%) of cactus supplied on a FB to the diet. The rejections of commercial feed and cactus were weighed on a daily basis to determine the consumption of both per lactation day.

The age of the cactus cladodes offered to the gilts was approximately 90 days (chemical composition shown in Table 1). The required cladode amount was cut manually every week. Thereafter, the cladodes were stored at 4 °C until they were given to the gilts. The cladodes were cut into pieces that measuring approximately 3 × 2 cm, and the amount required for each gilt was immediately added to their BD ratio at 8:00 h. This practice was performed daily during the experimental phase.

To evaluate the milk quality, 10 mL of milk was collected from each gilt on days 3, 11, and 17 of lactation. The milk was collected manually and randomly from the gilt’s nipples after an intramuscular injection of 2.0 mL of oxytocin^®^. Each sample was placed in a sterile container (100 mL capacity) and stored at 4 °C for further analysis (1 h post-milking) using Lactoscan^®^ equipment (Milkotronic Ltd., Nova Zagora, Bulgaria), which determined the lactose, protein, and fat contents. The piglets were weighed at birth (the weight of the piglets was determined as the piglets were born) and at weaning. The gilts were weighed on days 85 and 110 of gestation and at weaning. The following equation was used to estimate the gilt weight loss at weaning (GWLW, %):

(1) $$GWLW=100-\left(\frac{WGW*100}{WGPF}\right)$$

where WSW (kg) corresponds to the weight of the gilt at weaning and WSPF (kg) corresponds to the weight of the gilt post-farrowing. The weight of the post-farrowing gilt was estimated using the prediction equation of Mallmann et al. [17]. 

### 2.2. Blood Sampling 

On days 85 and 100 of gestation and on days 0 (farrowing day), 3, 7, 14, and 21 of lactation, six gilts per group were selected for preprandial (12 h fasting) blood sampling. The selection of the gilts for the monitoring of biochemical indicators was randomized. The six gilts selected from each group underwent continuous monitoring for sampling on the previously established days. A 10 mL blood sample was collected from the vena jugularis between 7:00 and 7:30 (one h before the start of the morning meal). Immediately after sampling, each blood sample was divided into two subsamples; 6 mL was placed in tubes with a serum cloth activator (for the glucose, triglyceride, and cholesterol analysis) and 4 mL was transferred into tubes with lithium heparin (for the insulin and osteocalcin analyses). The subsamples were stored at 4 °C until centrifugation. Subsequently, the tubes were centrifuged (1000× *g* for 10 min) and the serum were stored frozen at −20 °C until further analysis.

### 2.3. Blood Analyses 

The plasma glucose, triglyceride, cholesterol, insulin, and osteocalcin concentrations were determined. The determination of glucose, triglycerides, and cholesterol was carried out through enzymatic methods adapted in a Cobas C111 Mira (Roche, Basel, Switzerland) with the following reagents: GLUH2 (ref. 04 657 527 190, E.E.U.U.) (sensitivity: 2.0 mg·dL^−1^; intra- and inter-assay variation coefficients: <1.0% and <1.9%, respectively, at 128.0 mg·dL^−1^), TRIGL (ref. 04 657 594 190, E.E.U.U.) (sensitivity: 9.0 mg·dL^−1^; intra- and inter-assay variation coefficients: <8.0% and <14.0%, respectively, at 600 mg·dL^−1^), and CHOL2 (ref. 04 718 917 190, E.E.U.U.) (sensitivity: 10.0 mg·dL^−1^; intra- and inter-assay variation coefficients <10.0% and <12.0%, respectively, at 300 mg·dL^−1^). The insulin and osteocalcin levels were determined using commercial ELISA kits (Sigma-Aldrich, St. Louis, MO, USA). The sensitivities for each hormone were insulin: 4 µIU·mL^−1^ (intra- and inter-assay variation coefficients: <10% and <12%, respectively, at 47.5 µIU·mL^−1^); osteocalcin: 5 pg·mL^−1^ (intra- and inter-assay variation coefficients: <10% and <12%, respectively, at 90.5 pg·mL^−1^). 

### 2.4. Statistical Analyses 

The data were analyzed using a repeated measures ANOVA in PROC MIXED (SAS 9.4 Inst. Inc., Cary, NC, USA) [18]. The gilt represented the experimental unit in the model. The effects of the group, day, and their interaction were evaluated in terms of the feed intake; gilt weight; milk quality; piglet development; and plasma glucose, insulin, triglyceride, cholesterol, and osteocalcin concentrations. The model used was:

(2) $$Y_{ijkl}=\mu +G_{i}+C{\left(G\right)}_{j\left(i\right)}+D_{k}+G*D_{ik}+\varepsilon_{ijkl},$$

where *Y_ijkl_* = response variable: feed intake, gilt weight, milk quality, piglet development, and concentration of biochemical indicators evaluated; *µ* = constant common in the population; *G_i_* = fixed effects of the *i*-th group, with *i* = I, II, III, IV; *G(G*)*_j_*_(*i*)_ = random effect of the *j*-th gilt, nested within the *i*-th group, with *i* = I, II, III, IV; *D_k_* = fixed effects of the *k*-th day of lactation, with *k* = 1, 2, 3,…, 21; *G* * *D_ik_* = fixed effects of the interaction of the *i*-th group with the *k*-th day of lactation; *ε_ijkl_* = random effect associated with each observation (~NID = 0, σ^2^_e_).

The data of feed intake (per week) and the loss of body weight of the gilts were evaluated through ANOVA in PROC GLM (SAS 9.4 Inst. Inc., Cary, NC, USA). The effects of the group, week, and their interaction were evaluated. The model used was:

(3) $$Y_{ijk}=\mu +G_{i}+W_{j}+G*W_{ij}+\varepsilon_{ijk},$$

where *Y_ijk_* = response variable: feed intake (per week) and loss of body weight of the gilts; *µ* = constant common in the population; *G_i_* = fixed effects of the *i*-th group, with *I* = I, II, III, IV; *D_j_* = fixed effects of the *j*-th week of lactation, with *j* = 1 (day 1 to 7), 2 (day 8 to 14), 3 (day 15 to 21); *G* * *W_ij_* = fixed effects of the interaction of the *i*-th group with the *j*-th week of lactation; *ε_ijk_* = random effect associated with each observation (~NID = 0, σ^2^_e_).

Orthogonal polynomial contrasts were used to determine the linear and quadratic effects of increasing cactus levels on the feed and cactus intake. The differences between the groups were obtained by the least squares means method (LSmeans). Significant differences among the groups were considered at *p* < 0.05. The normality of the distribution and the homogeneity of variance for residuals were tested using PROC UNIVARIATE (SAS 9.4 Inst. Inc., Cary, NC, USA). In the case of non-normality, the parameters were normalized by *log* transformation prior to analysis to generate a normal distribution. 

Pearson’s correlations (PROC CORR; SAS 9.4 Inst. Inc., Cary, NC, USA) were determined between the average daily feed intake and cactus consumption and the concentrations of the biochemical parameters were evaluated. A principal component analysis (PCA) was performed using the FACTOR procedure of SAS (method = prin) with the Kaiser criterion—that is, an eigenvalue of ≥1.0 (mineigen = 1) [19]—to extract the principal components. No rotation method was applied to the principal components. Once Pearson’s correlation coefficients were established between the cactus consumption and feed intake, cactus consumption, and biochemical indicators, the quadratic and cubic regression were estimated. Each estimated regression equation was derived and equal to zero to determine the critical points. The results are presented as mean ±SE, and the differences were considered significant at *p* < 0.05.

## 3. Results

### 3.1. Gilt Performance 

**Figure 1:**
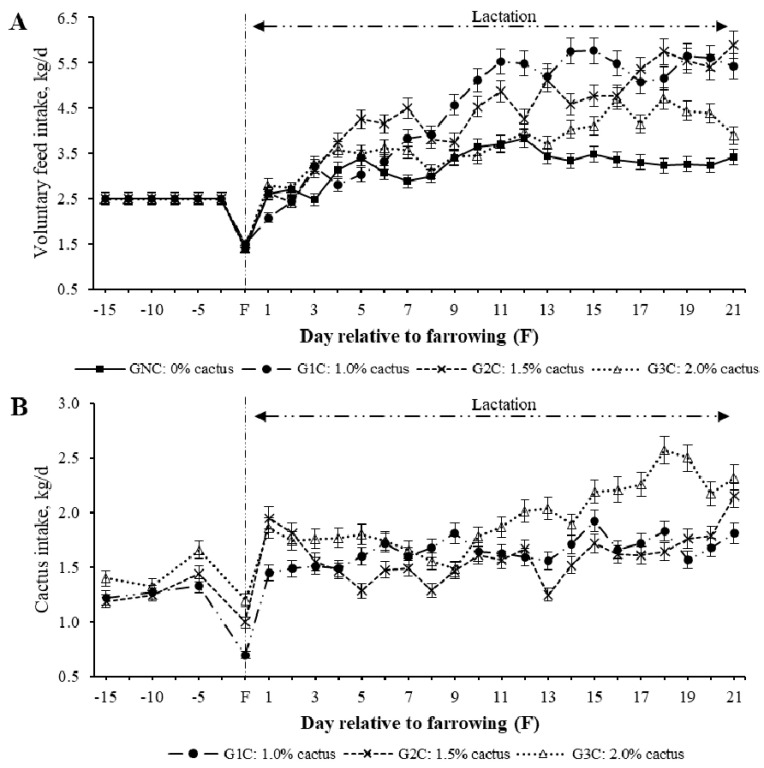
Least squares mean for the voluntary feed (**A**) and cactus (**B**) intake of gilts during lactation according to the group per day interaction.

The effect of cactus consumption was estimated by group, day, and group per day interaction. A group effect (*p* = 0.0003) was found on voluntary feed intake (VFI); the gilts subjected to G1C (4.50 kg/day) and G2C (4.38 kg/day) had the highest average VFI during lactation, while those subjected to GNC (3.26 kg/day) and G3C (3.77 kg/day) had the lowest average VFI (*p* < 0.05). Regarding the effect (*p* < 0.001) of the group per lactation day, the G1C and G2C had a higher VFI from the third day of lactation compared to the GNC (*p* < 0.05) (Figure 1). In the second week of lactation, the average VFI was equal (*p* > 0.05) between the GNC and G3C (Table 2). During the third week of lactation, the GNC had the lowest VFI compared to the groups that consumed cactus (*p* < 0.05) (Table 2).

Regarding the group effect (*p* = 0.0015) on cactus consumption, this varied owing to rejection, regardless of which of the three cactus levels (1.0%, 1.5%, and 2.0%) was added to the diet depending on the body weight of the gilts (Figure 1). The maximum (*p* < 0.05) average cactus consumption during lactation was in G3C [2.21 kg/day (1.2%)]. The average cactus consumption in the G1C and G2C was 1.65 and 1.78 kg·day^−1^, respectively (0.9% and 1.0%, respectively) (Table 2). 

The milk quality (fat, lactose, and protein content) was affected by the group (*p* < 0.05) as well as by the group per day interaction (*p* < 0.001). The highest fat, lactose, and protein contents in milk were found on day three of lactation in the GNC and the G1C (Table 3). On day 11 of lactation, no differences in the fat and protein content were found as a result of the group (Table 3). The lactose content was higher (*p* < 0.05) in the GNC and in the G1C on day 11 of lactation (Table 2). The lowest (*p* < 0.05) fat, lactose, and protein contents on day 17 of lactation were found in the G3C (Table 3).

The body weight loss of the gilts was affected by the group (*p* < 0.0001). The gilts of the GNC had higher (*p* < 0.05) body weight losses; these were followed by the gilts of the G3C (Table 3). There was no group effect on the weight of piglets at birth (*p* = 0.8207) and on the number of weaned piglets (*p* = 0.9741) (Table 3). The weight of the weaned piglets (day 21 of lactation) was affected (*p* = 0.0432), with piglets born to gilts of the group with 2.0% cactus having the lowest weight at weaning (Table 3).

### 3.2. Hormones and Metabolites

**Figure 2:**
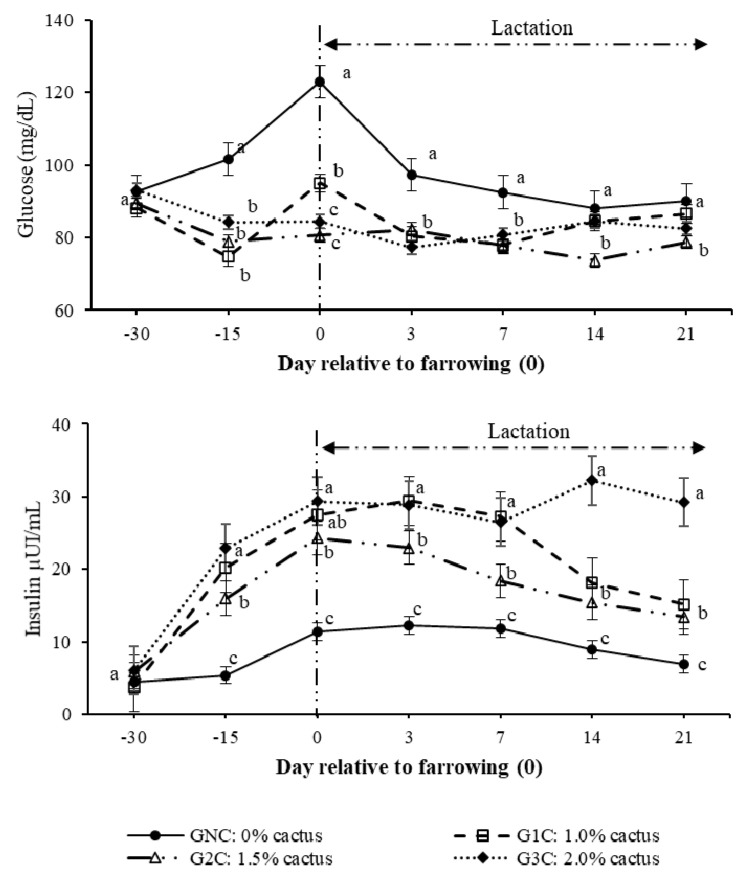
Plasma glucose and insulin concentrations as a result of the group and day. Different letters indicate a statistical difference (*p* < 0.05) among the groups for each evaluation day.

Regarding the effect (*p* < 0.0001) of the group per day interaction on the plasma glucose concentration, gilts in the control group had higher (*p* < 0.05) concentrations from day 100 of gestation to day seven of lactation (Figure 2). During lactation, the gilts that consumed cactus had plasma glucose concentrations of between 73.0 and 83.9 mg/dL (Figure 2). Regarding the effect (*p* < 0.0001) of the group per day interaction on the insulin concentration, the GNC had the lowest insulin concentrations (*p* < 0.05); these concentrations were constant from day 100 of gestation until day 14 of lactation, ranging between 8.9 and 11.2 µUI/mL. The G2C and G3C had higher (*p* < 0.05) insulin concentrations from day 100 of gestation until day seven of lactation compared to G1C. On day 14 of lactation, G1C and G2C had equal insulin concentrations (*p* > 0.05), which were lower (50.1% less) than those registered in G3C (Figure 2).

**Figure 3:**
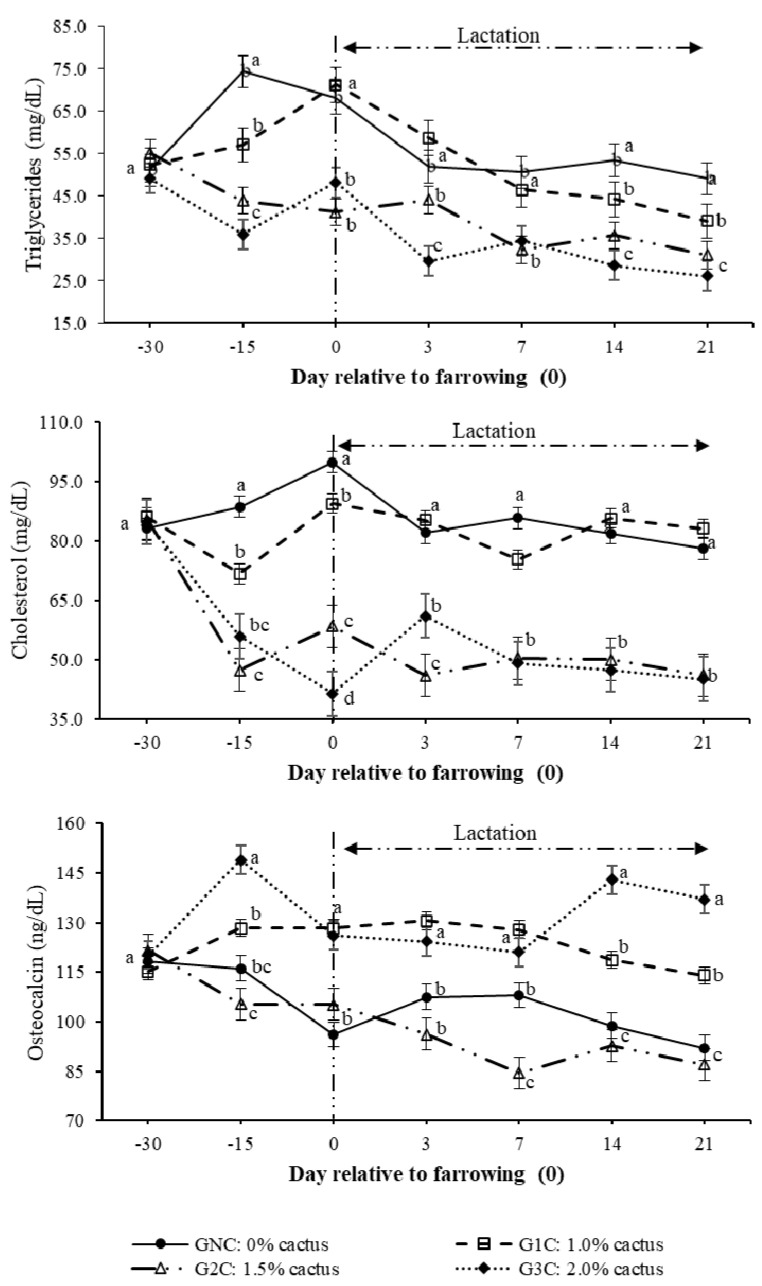
Plasma triglyceride, cholesterol, and osteocalcin concentrations as a result of the group and day. Different letters indicate a statistical difference (*p* < 0.05) among the groups for each evaluation day.

The highest (*p* < 0.05) triglyceride concentration resulting from the group per day interaction was observed on day 100 of gestation in the control group. The lowest triglyceride concentration was observed in the G2C and G3C (*p* < 0.05) (Figure 3). During lactation (days 3–14), the triglyceride concentration decreased in gilts of all groups; however, G2C and G3C had lower (*p* < 0.05) concentrations (Figure 3). On day 100 of gestation, cholesterol behaved in the same manner as triglycerides; its concentration was higher in the GNC and lower in the G2C and G3C (*p* < 0.05). From day 3 to day 14 of lactation, the cholesterol concentrations of the GNC and G1C showed the same tendency; these concentrations were higher (*p* < 0.05) compared to the G2C and G3C (Figure 3). 

Regarding the osteocalcin concentration that resulted from the effect (*p* < 0.0001) of the group per day interaction, this was the highest (*p* < 0.05) on day 100 of gestation in the G3C (Figure 3). During lactation, the highest (*p* < 0.05) osteocalcin concentration was observed in the G1C and G3C; however, on day 14 of lactation, the highest (*p* < 0.05) osteocalcin concentration was observed in the G3C (Figure 3). The greatest (*p* < 0.05) osteocalcin decrease was observed on day seven of lactation in the G2C (Figure 3).

### 3.3. Principal Component Analysis, Regression Models, and Optimal Cactus Level 

The PCA for the biochemical indicators resulted in the generation of two principal components (PC) that explained 72.19% of the variation (Table 4). The first component (PC 1) accounted for 36.85% of the total variability, and the concentrations of the indicators of energy metabolism and adiposity (glucose, total cholesterol, and triglycerides) were the main contributors to its formation; the concentrations of these biochemical indicators had a positive interrelationship, as their coefficients had the same sign. The second component (PC 2) explained 35.34% of the variability, and the greatest representation corresponded to biochemical indicators that affect glucose kinetics (Table 4).

The PC regression model was significant (*p* < 0.0001; R^2^ = 0.672) (Table 5). The lactation day was incorporated into the regression model because of the close relationship (r = 0.61; *p* < 0.0001) that existed with feed intake; the regression estimator (β) for this indicator was 0.099 (*p* < 0.0001). The estimated β values were −0.332 (*p* = 0.022) and −0.151 (*p* = 0.258) for PC 1 and PC 2, respectively (Table 5). Although the estimated β value for PC 2 was not significant, it was not eliminated from the model as PC 2 explained 35.34% of the variability (Table 4).

The regression estimators for feed intake and the biochemical indicators resulting from the cactus consumption are shown in Table 6. The orthogonal polynomial analysis of each indicator determined that the consumed cactus, which ranged from 0.92% to 1.23% (average 1.04%) and was offered to pre-farrowing gilts on an FB depending on their body weight, resulted in higher feed intake and plasma insulin and osteocalcin concentrations, as well as in lower plasma glucose, triglyceride, and cholesterol concentrations (*p* < 0.05) (Table 6).

## 4. Discussion

In sows, physiological processes such as insulin resistance [3,4], breastfeeding [7], and the increase in postpartum gonadotropins [8] are inherent processes of the species favoring productive deficiency in the current pig production systems as they affect directly the metabolic state of the sow, which, in turn, affects the voluntary feed intake during lactation [2]. This was confirmed in the gilts of the control group, as they were the ones with the lowest feed intake (Figure 1 and Table 2), while the concentrations of their metabolic indicators (glucose, cholesterol, and triglycerides) were higher compared to the groups that consumed cactus (Figure 2 and Figure 3).

The effect of cactus consumption on the feed intake and glycemic reduction in breeding sows has already been investigated [15,16,20]. However, in the present study, we observed that the higher the addition of *O. ficus-indica* (2.0%) according to the body weight of the gilts, the lower the feed intake (Figure 1 and Table 2); additionally, a 2.0% cactus addition resulted in lower cholesterol and triglyceride levels than a 1.0% cactus addition (Figure 3). This cactus effect has been attributed to the interactions among the different cactus components, such as its soluble fiber content (pectins), that interfere with carbohydrate metabolism [21,22] and reduce movement and glucose absorption [23], which, in turn, result in reduced blood glucose levels [23,24]. Likewise, the glycemia reduction can also be attributed to the phenols and flavonoids contained in *O. ficus-indica* [25,26]. Phenols have been associated with tissue sensitivity to insulin through their ability to scavenge free radicals [25]. Flavonoids promote the absorption of glucose by peripheral tissues [26].

Ordaz et al. [20] associated the reduction in glycemia in pregnant and lactating sows that consumed *O. ficus-indica* with osteocalcin increases. These authors suggest that the greater availability of Ca^2+^ in *O. ficus-indica* results in higher osteocalcin and insulin synthesis, which modulates the energy metabolism by reducing glucose, cholesterol, and triglyceride concentrations. It is possible that the content (316.6 mg/100 g, dry base) and bioavailability of Ca^2+^ from *O. ficus-indica* [27,28] affect the formation of the bone matrix by minimizing the use of endogenous Ca^2+^. This was reflected in the higher osteocalcin and insulin concentrations, lower glucose concentration, and higher feed intake (Figure 1, Figure 2 and Figure 3 and Table 2).

The lower weaning weight of the piglets of the G3C (Table 3) was associated with the higher cactus consumption (Table 2), which resulted in lower milk fat, lactose, and protein contents than the control or G1C (Table 3). Glucose is not only a precursor in the formation of fat in milk; it is also essential for the formation of lactose [29]. Therefore, the glycemia reduction in the gilts that consumed a greater cactus quantity resulted in a lower milk quality and piglet weight at weaning (Table 3). Likewise, the higher consumption of cactus by G3Cwas associated with a lower feed intake and greater body weight loss than in G1C and G2C (Figure 1 and Table 2 and Table 3). The higher weight loss that resulted from G3C (compared to G1C and G2C) was attributed to the high dietary fiber content consumed. Dietary fibers promote weight loss by reducing the energy intake by binding to dietary fats and making them unavailable for use in digestion [30]. A high dietary fiber consumption promotes a feeling of satiety [31], which reduces feed intake and promotes weight loss. The greater body weight loss of the control group sows compared to the G3C was independent of the similar feed intakes of the two groups. It has been reported [32] that the production of short chain fatty acids (propionic and acetic) as a result of the fermentation of fiber in the colon provides energy for maintenance and physiological processes. Therefore, the higher colonic fermentation of G3C compared to that of controls resulted in a reduction in their body weight loss (Table 3).

According to PC 1, the indicators of energy metabolism and adiposity (Table 4) affected the feed intake reduction, as, according to the PC regression estimators (Table 5), the β value of PC 1 was −332 (*p* = 0.022), which, in biological terms, indicates that for each unit of increase in the indicators that made up PC 1, there was a 332 g feed intake reduction. PC 2 represented the indicators that affect glucose kinetics—more specifically, insulin and osteocalcin (Table 4). Another mechanism that affects glucose kinetics and feed intake and is determined by osteoblasts is currently known [33]. This is associated with insulin [34] acting on osteoblasts and stimulating or inhibiting osteocalcin synthesis, which modulates insulin sensitivity [35]. It has been reported [36] that osteocalcin increases insulin secretion and the sensitivity to it through an increase in adiponectin, a hormone that stimulates the proliferation and improves the function of β cells, promotes a reduction in fatty tissue, and increases energy consumption. The results of the present study show that sows that consumed *O. ficus-indica* had higher osteocalcin levels (Figure 3), an observation that was also reported by Ordaz et al. [20].

Physiologically, the increased osteocalcin demand is associated with two processes through which the sow transitions: (1) bone resorption during the last third of gestation for the formation of the fetal skeleton and (2) the Ca^2+^ supply to milk [7]. However, a Ca^2+^ deficiency owing to the demand for fetal bone formation and milk production is associated with lower osteocalcin synthesis, which causes less insulin secretion and sensitivity [37]. Therefore, the feed intake modulation during lactation according to the PCA was positively governed by PC 2 (indicators that modulate glucose kinetics), which explained 35.34% of the variability, and negatively governed by PC 1 (indicators of energy metabolism and adiposity), which explained 36.85% of the variability (Table 4).

We estimated the critical points according to the regression equations of each biochemical indicator that made up each PC (Table 6). According to these critical points, we concluded that the optimal *O. ficus-indica* consumption should be between 0.90% and 1.23% (average 1.04%). A cactus consumption within this range resulted in a higher feed intake (4.20 kg); lower glucose (79.32 mg/dL), triglyceride (44.61 mg/dL), and cholesterol (66.03 mg/dL) concentrations; and higher insulin (25.05 µUI/mL) and osteocalcin (122.85 ng/mL) concentrations. Finally, there are some limitations that must be taken into account when interpreting the results of this study., regardless of existing reports [15,16,38] of the effects of the use of cactus (fresh basis) on the improvement in the indicators reproductive and productive of the sow and production costs. Cactus on a fresh basis has been used as a pilot test to evaluate its effects, since adding cactus on a fresh basis is a limitation in conventional pig production systems. This is the reason why more research must be carried out on how to industrialize the cactus to provide it in conventional diets. However, this research provides valuable information on the inclusion level of cactus in the feeding of breeding sows.

## 5. Conclusions

The voluntary feed intake of sows during lactation does not increase with an increasing *O. ficus-indica* consumption. The addition of 1.04% *O. ficus-indica*, depending on the body weight of the gilts, is optimum, as it results in the best biochemical indicator (glucose, insulin, cholesterol, triglycerides, and osteocalcin) values, which lead to a higher voluntary feed intake during lactation. However, this technology may be beneficial to farmers who manage less industrialized systems of swine production. More research should be done in which a greater number of experimental units are used. Likewise, the developing new technologies for processing cactus in order to facilitate its use in conventional swine production systems should be investigated. 

## Figures and Tables

**Table 1:** Ingredients and nutritional composition of diets.

Item	Basal Diets	
Ingredients (g/kg)	Gestation	Lactation
Sorghum	824.0	649.5
Soy paste	60.0	100.0
Canola paste	61.2	185.2
Orthophosphate	11.8	5.3
Calcium carbonate	14.0	12.4
Soy oil	22.0	38.5
Lysine	1.2	2.5
Methionine + Cysteine	0.9	1.5
Salt	3.0	3.0
Vitamin and mineral premix ^a^	2.0	2.5
Nutrient levels (%)		
Metabolizable energy (MJ/kg)	13.6	13.9
Crude protein	15.5	18.6
Lysine	0.79	0.95
Methionine + cysteine	0.43	0.59
Calcium	1.4	1.2
Total phosphorus	0.64	0.67
Available phosphorus	0.33	0.46
Nutrient levels of *Opuntia ficus-indica* (%)		
Crude protein	5.6	
Crude fat	0.2	
Fiber	28.8	
Humidity	88.6	
Ashes	24.5	
Nitrogen-free elements	40.8	
Mucilage (g/300 g dry base)	2.6	

^a^ Contribution per kg of feed: Cu 30 mg; Fe 160 mg; Zn 160 mg; Mn 55 mg; Se 0.5; Cr 0.2 mg; Vitamin A 14.200 IU; Vitamin D_3_ 2800 IU; Vitamin E 125 mg; Vitamin K_3_ 5 mg; Vitamin B_1_ 2.4 mg; Vitamin B_2_ 8.7 mg; Vitamin B_6_ 4.5 mg; Vitamin B_12_ 0.05 mg; Pantothenic acid 35 mg; Acid folic 6 mg.

**Table 2:** Effect of the cactus inclusion levels on the feed and cactus intake in gilt pigs during late gestation and lactation.

Item	Feed Intake				SEM	*p*-Value ^&^	
	GNC: 0% Cactus	G1C: 1% Cactus	G2C: 1.5% Cactus	G3C: 2% Cactus		L	Q
*Gestation*							
Day 85 to 115	2.24	2.22	2.23	2.24	0.05	0.8641	0.6518
*Lactation*							
Day 1 to 7	3.05	3.12	3.54	3.40	0.10	0.0015	0.2975
Day 8 to 14	3.46	5.08	4.38	3.60	0.10	0.5622	<0.0001
Day 15 to 21	3.32	5.41	5.21	4.31	0.10	<0.0001	<0.0001
Day 1 to 21	3.29	4.59	4.38	3.76	0.07	<0.0001	0.0461
	**Cactus Consumption**						
*Gestation*							
Day 85 to 115	--	1.12	1.21	1.41	0.04	0.0083	0.2318
*Lactation*							
Day 1 to 7	--	1.60	1.55	2.38	0.07	0.0021	0.1195
Day 8 to 14	--	1.71	1.57	1.97	0.06	0.0715	0.0172
Day 15 to 21	--	1.74	1.65	2.23	0.06	0.0109	0.0037
Day 1 to 21	--	1.69	1.59	2.19	0.03	<0.0001	<0.0001

^&^*p*-values are from orthogonal polynomial contrasts: L, linear; Q, quadratic.

**Table 3:** Least squares mean for the body weight loss of the gilts as well as the piglet development according to the feeding scheme and day.

Item	Day (D)	Groups (G)				SEM	*p*-Value		
		GNC: 0% Cactus	G1C: 1% Cactus	G2C: 1.5% Cactus	G3C: 2% Cactus		G	D	G * D
Gilts BW (kg)									
Day 85 of gestation		179.71	185.53	181.42	180.91	0.34	0.0836	--	--
Day 110 of gestation		191.32	196.38	190.33	190.72	0.34	0.0609	--	--
Post-farrowing ^&^		174.52	178.78	173.20	173.81	0.34	0.0813	--	--
Weaning		164.22 ^a^	176.91 ^b^	171.11 ^c^	169.25 ^c^	0.34	<0.0001	--	--
Gilt LBW (%)		5.90 ^a^	1.05 ^b^	1.21 ^b^	2.62 ^c^	0.15	0.0415	--	--
Milk quality (%)									
	3	12.5 ^a^	11.6 ^a^	9.3 ^b^	9.0 ^b^	0.39			
Fat	11	6.6 ^a^	6.1 ^a^	6.2 ^a^	6.0 ^a^	0.39	0.0419	<0.0001	<0.0001
	17	5.8 ^a^	5.0 ^b^	5.1 ^b^	4.7 ^c^	0.39			
	3	7.1 ^a^	7.3 ^a^	6.1 ^b^	5.7 ^b^	0.39			
Lactose	11	6.4 ^a^	6.2 ^ab^	5.8 ^b^	5.6 ^b^	0.39	0.0218	<0.0001	<0.0001
	17	6.3 ^a^	6.2 ^a^	5.9 ^b^	5.8 ^b^	0.39			
	3	6.1 ^a^	6.1 ^a^	4.6 ^b^	4.5 ^b^	0.39			
Protein	11	5.3 ^a^	5.9 ^a^	5.5 ^a^	5.0 ^a^	0.39	0.0346	<0.0001	<0.0001
	17	5.4 ^a^	5.7 ^a^	5.6 ^a^	4.6 ^b^	0.39			
Piglet weight at birth (kg)		1.6	1.5	1.5	1.5	0.12	0.8207	--	--
Piglet weight at weaning (kg)		5.9 ^a^	5.7 ^a^	5.5 ^a^	4.8 ^b^	0.15	0.0432	--	--
Weaned piglets		9.6	9.5	9.7	9.0	0.13	0.9741	--	--

BW = body weight; LBW = loss of body weight. ^&^ Estimated according to the prediction equations established by Mallann et al. (2018). ^a,b,c^ Different literals indicate statistical differences (*p* < 0.05) within the row.

**Table 4:** Principal component analysis. Eigenvalues, explained and cumulative variance, loadings of the variables for the first two principal components.

	Principal Component	
	1	2
*Variance explained*		
Eigenvalue	1.842	1.767
Variance (%)	36.850	35.342
Cumulative (%)	36.850	72.192
*Factor loadings*		
Glucose	0.614	0.050
Total cholesterol	0.925	−0.217
Triglycerides	0.819	−0.222
Insulin	−0.197	0.755
Osteocalcin	−0.011	0.997

**Table 5:** Regression estimators to establish the effect of the components that describe the feed intake in lactating gilts.

Model	Non-Standardized Coefficients		Standardized Coefficients	t	*p*-Value	Confidence Interval for β (95.0%)	
	Β	SD	β			LL	UL
Constant	2.711	0.223		12.164	<0.0001	2.267	3.155
Day	0.099	0.019	0.495	5.203	<0.0001	0.061	0.137
PC 1	−0.332	0.141	−0.222	−2.343	0.022	−0.613	−0.050
PC 2	−0.151	0.139	−0.102	−1.092	0.278	−0.427	0.125

LL = lower limit; UL = upper limit.

**Table 6:** Regression estimators for feed intake and biochemical indicators according to cactus consumption.

	Item ^&^	Regression Estimators *				Critical Point		R^2^
						CL (%)	IV	
		Intercept	β1	β2	β3			
	Feed intake (kg)	3.13 (<0.0001)	2.36 (<0.0001)	−1.28 (<0.0001)	-- --	0.92	4.20	0.26
**PC 1**	Glucose (mg/dL)	93.97 (<0.0001)	−22.32 (<0.0001)	8.62 (0.0040)	-- --	1.23	79.32	0.23
	Triglycerides (mg/dL)	53.13 (<0.0001)	−47.58 (0.0010)	70.46 (0.0018)	−31.40 (0.0011)	0.98	44.61	0.31
	Cholesterol (mg/dL)	81.92 (<0.0001)	−85.87 (<0.0001)	122.40 (<0.0001)	−52.43 (<0.0001)	1.02	66.03	0.28
**PC 2**	Insulin (µUI/mL)	13.87 (<0.0001)	20.78 (<0.0001)	−9.69 (<0.0001)	-- --	1.07	25.05	0.39
	Osteocalcin (ng/mL)	105.39 (<0.0001)	33.88 (0.0079)	−16.43 (0.0554)	-- --	1.03	122.85	0.21

CL = cactus level; IV = indicator value. ^&^ Estimates for each indicator were determined according to the percentage of cactus consumed. * estimator (*p*-value).
